# Reply: Dietary acrylamide and cancer risk: additional data on coffee

**DOI:** 10.1038/sj.bjc.6601180

**Published:** 2003-08-12

**Authors:** L A Mucci, P W Dickman, G Steineck, H O Adami, K Augustsson

**Affiliations:** 1 Department of Medical Epidemiology and Biostatistics, Karolinska Institutet, Box 281, SE 171 77 Stockholm, Sweden; 2 Department of Epidemiology, Harvard School of Public Health, 677 Huntington Avenue, 9th Floor, Boston, MA 02115, USA; 3 Department of Oncology and Pathology, Clinical Cancer Epidemiology, Karolinska Institutet, SE 171 76 Stockholm, Sweden

**Sir**,

We appreciate the comments of Drs Hagmar and Törnqvist (2003) on our article assessing dietary acrylamide and cancer risk (Mucci *et al*, 2003). We take this opportunity to clarify some issues and to present data from additional analyses.

We undertook the original investigation in light of claims by the Swedish National Food Administration that acrylamide in foods could have global impacts on cancer incidence rates. In spite of the potential limitations of the study design, our data are reassuring that acrylamide seems unlikely to be responsible for a major fraction of these cancers. As stated in our discussion, however, additional data are needed before a global assessment of any risks of dietary acrylamide can be undertaken in relation to other cancer sites and neurological diseases.

The reliance on toxicological risk assessment models employed by Hagmar and Törnqvist may be questionable. Estimates of human cancer risk were extrapolated from animal models, given doses of acrylamide several fold higher than those to which humans are exposed (International Agency for Research on Cancer (IARC), 1994). We think that animal data must be generalized to humans with caution. This sentiment is reflected by IARC (2002) who considers an agent as definitely carcinogenic to humans when there is sufficient evidence from studies in humans (i.e. epidemiological evidence) and only ‘exceptionally’ in other situations.

The authors present evidence from power analyses on the substantial sample size needed to detect an effect of acrylamide on human cancer risk. Notwithstanding the limitations of the risk assessment models, the authors determined an expected relative risk of 1.05 for the highest *vs* lowest dose. An effect estimate of this size is almost impossible to determine in any observational study. Indeed, not even a randomised clinical trial would have the power to detect this effect. The scientific methods to study such a small effect currently do not exist, and beg the question of how to best proceed to address the question of acrylamide and cancer. In addition, we must ask whether a relative risk of this size warranted the public health alarm that was generated when the findings of acrylamide in food were first announced.

## 

### Additional data

Since our study was published, new data have come available on acrylamide content in additional food items. In particular, acrylamide has been detected in coffee. Although the range of exposure (∼8 μg kg^−1^) is lower than other items, coffee may account for a substantial proportion of total dose because of the frequency of consumption. We present updated data from the original case–control study, using a similar methodology.

Coffee consumption was common in this Swedish population, with 23.5% of controls consuming four or more cups of coffee per day. The daily mean (standard error) dietary acrylamide dose (*μg*) increased with the addition of coffee data: 34.0 (0.6) for controls, 34.8 (0.6) for colorectal, 36.8 (1.0) for bladder, and 34.5 (1.4) for kidney cancers. Crisp breads (28%) and coffee intake (20%) contributed to the largest sources of acrylamide in the diet among controls (Figure 1

**Figure 1:**
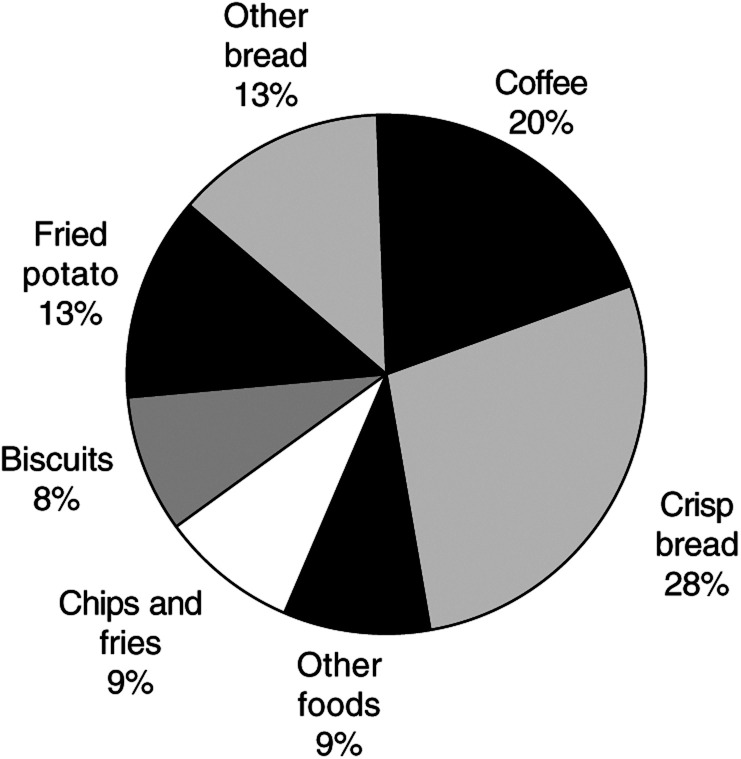
Major sources of acrylamide in investigated foods in the Swedish diet, among controls.

). Quartiles of dietary acrylamide dose were calculated based on the distribution among the controls. Adjusting for potential confounders, the risk of colorectal (Figure 2A

**Figure 2:**
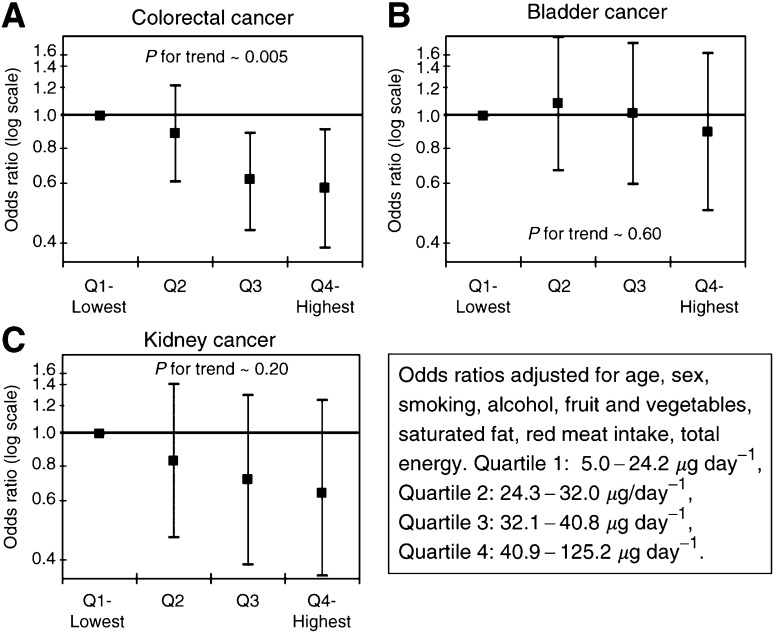
Quartiles of daily dietary acrylamide dose and risk of cancer of the (**A**) large bowel, (**B**) bladder and (**C**) kidney.

) and kidney cancers (Figure 2C) decreased with increasing acrylamide dose. The apparent protective effect of acrylamide parallels the lower risk associated in this study with crisp breads and for coffee, a finding consistently observed in the literature (Ekbom, 1999). The relative risk estimate of acrylamide and bladder cancer was essentially null (Figure 2B).

Expanding the range of exposure and achieving a more complete estimate of acrylamide intake, there remains no evidence of an excess risk of the three studied cancers in relation to acrylamide, and provides further reassurance that acrylamide in diet does not appear to be responsible for a major fraction of these three cancers.

## References

[bib1] Ekbom A (1999) Review: substantial coffee consumption was associated with a lower risk of colorectal cancer in the general population. Gut 44: 597 10205191 10.1136/gut.44.5.597 PMC1727503

[bib2] Hagmar L, Törnqvist M (2003) Inconclusive results from an epidemiological study on dietary acrylamide and cancer. Br J Cancer 89(4): 774–775 12915892 10.1038/sj.bjc.6601016 PMC2376907

[bib3] International Agency for Research on Cancer (IARC) (1994) IARC Monographs on the evaluation of Carcinogen Risk to Humans: Some Industrial Chemicals, No. 60, Lyon: International Agency for Research on Cancer Press

[bib4] International Agency for Research on Cancer (IARC) (2002) IARC Monographs on the Evaluation of Carcinogenic Risks to Humans.Vol 80. Non-Ionizing Radiation Part 1: Static and Extremely Low Frequency (ELF) Electric and Magnetic Fields, p. 26, Lyon, France: IARC Press PMC5098132 12071196

[bib5] Mucci LA, Dickman PW, Steineck G, Adami H-O, Augustsson K (2003) Dietary acrylamide and cancer of the large bowel, kidney, and bladder: absence of an association in a population-based study in Sweden. Br J Cancer 88: 84–89 12556964 10.1038/sj.bjc.6600726 PMC2376776

